# Dengue virus in *Aedes aegypti* and *Aedes albopictus* in urban areas in the state of Rio Grande do Norte, Brazil: Importance of virological and entomological surveillance

**DOI:** 10.1371/journal.pone.0194108

**Published:** 2018-03-13

**Authors:** Arlinete S. Medeiros, Diego M. P. Costa, Mário S. D. Branco, Daíse M. C. Sousa, Joelma D. Monteiro, Sílvio P. M. Galvão, Paulo Roberto M. Azevedo, José V. Fernandes, Selma M. B. Jeronimo, Josélio M. G. Araújo

**Affiliations:** 1 Post-GraduateHealth Program, Health Sciences Center, Federal University of Rio Grande do Norte, Natal, Rio Grande do Norte, Brazil; 2 Laboratory of Molecular Biology for Infectious Diseases and Cancer, Department of Microbiology and Parasitology, Biosciences Center, Federal University of Rio Grande do Norte, Natal, Rio Grande do Norte, Brazil; 3 Laboratory of Virology, Institute of Tropical Medicine, Federal University of Rio Grande do Norte, Natal, Brazil; 4 Department of Statistics, Federal University of Rio Grande do Norte, Natal, Brazil; 5 Immunogenetics Laboratory, Department of Biochemistry, Biosciences Center, Federal University of Rio Grande do Norte, Natal, Brazil; 6 Laboratory of Complex Diseases, Institute of Tropical Medicine, Federal University of Rio Grande do Norte, Natal, Brazil; 7 Institute of Science and Technology of Tropical Diseases, Brasília, Brazil; Instituut voor Tropische Geneeskunde, BELGIUM

## Abstract

**Background:**

Vector control remains the sole effective method to prevent dengue virus (DENV) transmission, although a vaccine for dengue has recently become available and testing of its efficacy and coverage is being performed in multiple places. Entomological surveillance is a key factor in alerting authorities to possible outbreaks, but until now natural DENV infection of mosquito populations has been scarcely used as an early warning system to monitor fluctuating prevalence of infected mosquitoes. The purpose of this study was to determine the burden of adult and larval/pupae of *Aedes aegypti* and *Aedes albopictus* with DENV in urban areas in the state of Rio Grande do Norte, Brazil.

**Methodology/Principal findings:**

Immature insect forms (larvae and pupae) were collected from April 2011 to March 2012, whereas the collection of adults was conducted along 3 years: May 2011 to April 2014. Total RNAs of the samples were extracted and the nested reverse transcriptase PCR assay for detecting and typing DENV was performed. Of the 1333 immature insects collected during the study period, 1186 (89%) were *A*. *aegypti* and 147 (11%) *A*. *albopictus*. DENV-4 was identified in pools of *A*. *aegypti* larvae. The rate of DENV infection in immature *A*. *aegypti* was expressed as MIR = 3.37. DENV wasnot detected in immature *A*. *albopictus*. A total of 1360 adult female mosquitoes of the *Aedes* genus were captured from May 2011 to April 2014. Of this total, 1293 were *A*. *aegypti* (95%) and 67 were *A*. *albopictus* (5%). From the 130 pools studied, 27 (20.7%) were positive for DENV. DENV-1 was identified in 2/27 (7.4%) pools; 1of *A*. *albopictus* and 1 of *A*. *aegypti*. DENV-2 was identified in only 1/27 (3.7%) *A*. *aegypti* pools. DENV-4 was the most prevalent, identified in 24/27 (88.8%) of the positive pools, with 19 being of *A*. *aegypti* and 5 of *A*. *albopictus* pools. The minimum infection rate for adults of the *Aedes* genus was 19.8, considering both *A*. *aegypti* and *A*. *albopictus*.

**Conclusions/Significance:**

This work represents the most complete study to date on the interaction between dengue viruses and *Aedes* mosquitoes in the State of Rio Grande do Norte, and raises important questions about a possible role of *A*. *albopictus* in the transmission of dengue virus in Brazil.

## Introduction

Dengue virus in humans produces a wide symptomatic spectrum ranging from a mild clinical profile to dengue with warning signs, and can evolve to severe dengue. Factors related to outcome are usually associated with age, secondary infection, immunologic status, dengue serotype, orgenotype [1]. There is significant global diversity among dengue virus (DENV) strains. The four serotypes of DENV (DENV-1, DENV-2, DENV-3, and DENV-4) belong to the genus *Flavivirus* of the family *Flaviviridae*. They are genetically distinct, but cause similar diseases and share epidemiological features [2].

In the past 50 years, the incidence of dengue fever (DF) has increased 30-fold with increasing geographic expansion into new countries [3], including Brazil which had no reports of dengue cases for almost 40 years because of the successful elimination of *Aedes aegypti* attained in the 50’s. However, the virus was reintroduced in early 80’s in Brazil and rapidly spread all over. The majority of annual dengue notifications in the Americas are now from Brazil [4,5]. Dengue has been a major public health problem in the State of Rio Grande do Norte (RN) and other states in Brazilfor over twenty years. The first laboratory-confirmed cases in RN occurred in 1994. All four DENV serotypes have been found, and the frequencies of these serotypes within the population vary by geographic region of the state and year [6]. A total of 32,328 dengue cases and 40 deaths were reported in Natal Cityfrom 2011 to 2014, and within this period the highest number of cases occurred in 2012 (S1 Fig).

Transmission of DENV largely occurs from infected mosquitos to humans, defined as horizontal transmission, and it is largely considered the major route by which the virus remainsin an area. However, vertical transmission within the mosquito (infected female mosquito to infected offspring) has been suggested as a mechanism that ensures conservation of the virus during conditions that would be adverse for horizontal transmission (i.e. harsh winters, inter-epidemic stages) [7], and potentially influence on the epidemiology of dengue infection [8], thus leading to persistence of the virus without apparent need of human infection. Vertical transmission has been reported in *A*. *aegypti* under experimental [9,10] and natural conditions in different locationsin the world such as Myanmar [11], Mexico [12], Brazil [13] and Cuba [14].

Like *A*. *aegypti*, *Aedes albopictus* has now been implicated as an important vector for DENV [15–17], Chikungunya virus (CHIKV), and potentially Zika virus (ZIKV) transmission in African and European countries [15,18–20], and in mainland India [21]. More recently, CHIKV and ZIKV have become highly endemic in Latin America after their introduction in Brazil, and these viruses have rapidly spread worldwide.

The monitoring of dengue viruses in immature and adult mosquitoes represents an important strategy for identifying the beginning of the epidemic period and direct control actions, especially in difficult-to-access areas [22,23]. Therefore, the purpose of this study was to determine the incidence of adult and larval/pupae of *A*. *aegypti* and *A*.*albopictus* with DENV in urban areas in the state of Rio Grande do Norte, Brazil, in order to estimate the potential magnitude of dengue infection persistence in these insects.

## Methods

### Study area

**Fig 1:**
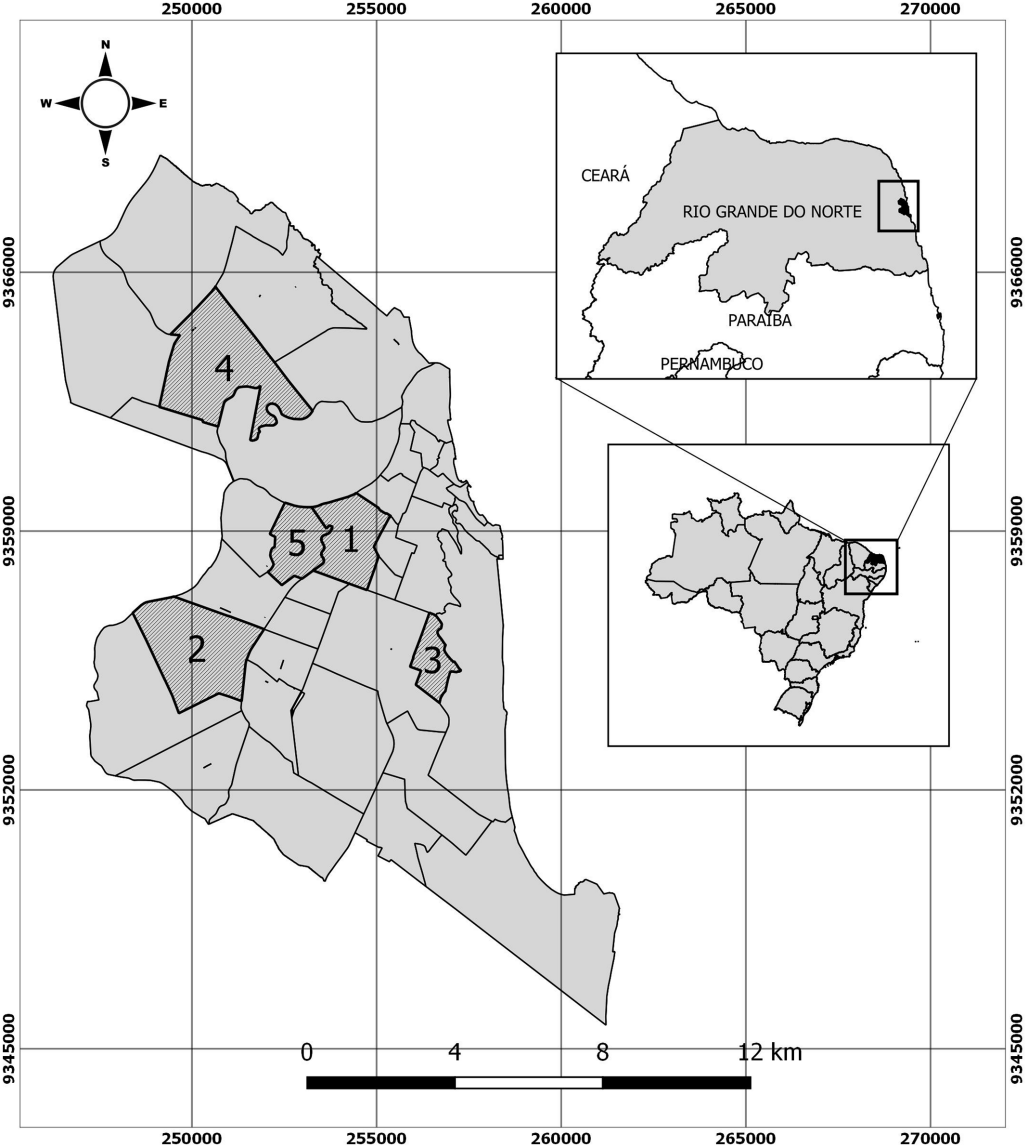
Study area. Map with the location point in the State of Rio Grande do Norte, Brazil. Areas where mosquitoes, larvae and pupae were collected. 1- Alecrim, 2- Felipe Camarão, 3- Nova Descoberta, 4- Potengi, and 5- Quintas. Note: This figure was created specifically for this manuscript by QGIS program. QGIS is a free and open-source cross-platform desktop geographic information system (GIS) application.

Natal has a total population of 885,180 people and is the capital of Rio Grande do Norte State in northeastern Brazil, located at5° 46'45.33"S, 35° 12'03.33"O [24]. The city has a total area of170 square kilometers (66 square miles) and has a tropical climate with a short rainy season, warm temperatures and high relative humidity throughout the year. Traps for insect captures were placed in tire repair sites and scrap metal recycling sites, selected in five urban areas of the town: Alecrim (area 1) (5° 47'52.75"S, 35° 13'03.30"O), Felipe Camarão (area 2) (5° 49'26.78"S, 35° 15'11.87"O), Nova Descoberta (area 3) (5° 49'23.74"S, 35° 11'58.28"O), Potengi (area 4) (5° 45'04.09"S, 35° 15'18.58"O), andQuintas (area 5) (5° 47'48.09"S, 35° 14'02.68"O) (Fig 1).

### Collection of Culicidae

The immature insect forms (larvae and pupae) were collected (*N* = 1333) from April 2011 to March 2012, whereas the collection of adults (*N* = 1360) was conducted along 3 years: May 2011 to April 2014. The collections were carried out in a tire repair shops located in each district of the city. In addition, a metal recyclingshop was randomly selected in each area. These sites were public. The immature samples were collected with the aid of a "shell" or Pasteur pipette from the breeding sites. The adults were collected with a castrotrap. The samples were then transferred to plastic containers and sent to the Laboratory of Entomology of the Public Health Secretary at of Rio Grande do Norte (SESAP/RN) for species identification. Adults were placed in a freezer for five minutes to be anesthetized, subsequently placed in petri dishes and then taken to an entomological loupe for identification and to separate females. The females were then placed in 2mL microtubes with mosquito number information, species, place and date of collection, and immediately stored in a freezer at -70°C.

### Preparation of samples

Pools of up to 40 immature (larvae and pupae) or adult female mosquitoes were macerated using plastic micropistilles in 500 μL of Leibovitz L15 medium (GIBCO-BRL, Gaithersburg, MD, USA) containing 2% fetal bovine serum, and centrifuged at 2500×g for 20 min at 4°C to sediment the carcasses. The supernatant was divided into 2 aliquots and stored at -70°C until use.

### RNA extraction and nested reverse transcriptase PCR assay

Total RNAs of the samples were extracted using the QIAmp Viral Mini Kit (QIAGEN, Inc., Valencia, USA) following the protocol described by the manufacturer. RNA was reverse transcribed to obtain the cDNA of each sample. cDNA was submitted to amplification using classical Nested reverse transcriptase PCR assayas described by Lanciotti et al. [25] for detecting and typing dengue viruses, and then the minimum viral infection rate was determined using the Minimum Infection Rate (MIR). This index is calculated by dividing the number of infected pools per species by the total number of mosquitoes tested for each species, multiplied by 1,000 [26]. Lastly, the proportion comparison test was performed using the Statistica 7.0 program, and a p-value ≤ 0.05 was considered as statistically significant.

## Results

### DENV infection in immature *Aedes aegypti* and *Aedes albopictus*

Of the 1333 immature larvae and pupae collected during the study period from the different locations in Natal, Brazil, 1186 (89%) were *A*. *aegypti* and 147 (11%) *A*. *albopictus*. A higher number of larvae were collected in August and September 2011 post-rainy season, which presented a mean precipitation of 348.5 mm and an average relative humidity of 83.5. One of the geographic areas (Felipe Camarao) had a higher incidenceof insects (Tables 1 and 2). DENV-4 was identified in *A*. *aegypti* larvae pools in the areas of Alecrim, Felipe Camarão and Nova Descoberta. The rate of DENV infection in *A*. *aegypti* was expressed as MIR = 3.37 (Table 1). The DENV was not detected in *A*. *albopictus* larvae or pupae in this study (Table 2).

**Table 1:** Minimum infection rate (MIR) for dengue virus in *Aedes aegypti* larvae and pupae collected in urban areas in Natal, Rio Grande do Norte, Brazil.

*Aedes aegypti*							
Sampling Area	Number of samples	Number of pools	Positive Pools	DENV	MIR	Proportion of positives	*p-value*
Alecrim	338	8	1	DENV-4		0.0029	0.1611
Felipe Camarão	270	13	2	DENV-4		0.0074	0.0787
Nova Descoberta	229	12	1	DENV-4	**3.37**	0.0043	0.1605
Potengi	210	6	0	-		-	-
Quintas	139	7	0	-		-	-
**Total**	**1,186**	**46**	**4**	**DENV-4**		**0.0034**	0.0223*

*Statistically significant.

**Table 2:** Dengue virus in *Aedes albopictus* larvae and pupae collected in urban areas in Natal, Rio Grande do Norte, Brazil.

*Aedesalbopictus*							
Sampling Area	Number of samples	Number of pools	Positive Pools	DENV	MIR	Proportion of positives	*p-value*
Alecrim	0	0	0	-		-	-
Felipe Camarão	74	5	0	-		-	-
Nova Descoberta	32	4	0	-	-	-	-
Potengi	19	2	0	-		-	-
Quintas	22	2	0	-		-	-
**Total**	**147**	**13**	**0**	-		-	-

### Research on DENV in *Aedes aegypti* and *Aedes albopictus* adults

A total of 1360 adult female mosquitoes of the *Aedes* genus were captured from May 2011 to April 2014. Of this total, 1293 were *A*. *aegypti* species (95%) and 67 were *A*. *albopictus* (5%) (Tables 3 and 4). The average of the three years of study revealed a higher number of adults collected in the months of July and August, right after the rainy season. From the 130 pools studied, 27 (20.7%) were positive for DENV. DENV-1 was identified in 2/27 (7.4%) pools, 1/2 (50%) being of *A*.*albopictus* in Nova Descoberta in 2011, and 1/2 (50%) of *A*. *aegypti* in Alecrimin 2013. DENV-2 was identified in only 1/27 (3.7%) *A*. *aegypti* pools in Potengi in August 2012. DENV-4 was the most prevalent, being identified in 24/27 (88.8%) of the studied pools; 19/24 (79.1%) being of *A*. *aegypti*, and 5/24 (20.8) of *A*. *albopictus* pools. DENV-4 was identified in all five areas studied from October 2011 to February 2014. It was possible to detect the presence of DENV-1 in a sample of only one *A*. *albopictus* individual. The same was observed for DENV-4, where this serotype was identified in 7 samples (6 *A*. *aegypti* and 1 *A*. *albopictus*) of only one mosquito.

**Table 3:** Minimum infection rate (MIR) for dengue virus in *Aedes aegypti* adults collected in urban areas in Natal, Rio Grande do Norte, Brazil.

*Aedes aegypti*							
Sampling Area	Number of samples	Number of pools	Positive Pools	DENV	MIR	Proportion of positives	*p-value*
Alecrim	250	32	1	DENV-1		0.0320	0.0023*
			7	DENV-4			
Felipe Camarão	442	36	5	DENV-4		0.0113	0.0126*
Nova Descoberta	0	0	0	-	**16.2**	-	-
Potengi	492	32	1	DENV-2		0.0122	0.0071*
			5	DENV-4			
Quintas	109	11	2	DENV-4		0.0183	0.0787
**Total**	**1,293**	**111**	**21**	**DENV-1, 2, 4**		**0.0162**	0.0000*

*Statistically significant.

**Table 4:** Dengue virus in *Aedes albopictus* adults collected in urban areas in Natal, Rio Grande do Norte, Brazil.

*Aedes albopictus*							
Sampling Area	Number of samples	Number of pools	Positive Pools	DENV	MIR	Proportion of positives	*p-value*
Alecrim	0	0	0	-		-	-
Felipe Camarão	16	10	3	DENV-4		0.1875	0.0394*
Nova Descoberta	15	3	1	DENV-1		0.1333	0.0772
			1	DENV-4	-		
Potengi	36	6	1	DENV-4		0.0278	0.1586
Quintas	0	0	0	-			
**Total**	**67**	**19**	**6**	**DENV-1, 4**		**0.0896**	0.0067*

*Statistically significant.

### Minimum infection rate of dengue viruses in *Aedes aegypti* and *Aedes albopictus* adults

The minimum infection rate for adults of the *Aedes* genus was 19.8, considering *A*. *aegypti* and *A*. *albopictus*. Considering only *A*. *aegypti*, the MIR was 16.2. In analyzing the infection individually by DENV-1, DENV-2 and DENV-4 in *A*. *aegypti*, the MIR was 0.7, 0.7 and 14.6, respectively. It was not possible to calculate the MIR of the *A*. *albopictus*, since less than 1000 individuals of this species were collected.

## Discussion

The state of Rio Grande do Norte (RN), Brazil, has reported cases of dengue fever since October 1994, when the first autochthonous cases were reported in the municipality of Assú after an out-of-season carnival in the region. Since then, this state has demonstrated an endemic profile for dengue, with increasingly frequent and intense epidemic years due to several aspects, such as an emergence or reemergence of DENVserotypes. In 2015, Zika and Chikungunya emerged with great impact, including an increase in Microcephaly and Guillain-Barre Syndrome cases. Vector control remains the sole effective method to prevent DENV transmission, although a vaccine for dengue has recently become available and testing of its efficacy and coverage isbeing performedin multiple places. Entomological surveillance is a key factor in alerting authorities to possible outbreaks, but until now natural DENV infection of mosquito populations has been scarcely used as an early warning system to monitor fluctuating prevalence of infected mosquitoes [14]. We have focused ondeterming the magnitude of horizontal infection in DENV vector transmitters *A*. *aegypti* and *A*. *albopictus*. This study provides the first description of the natural transovarian transmission of dengue virus in *A*. *aegypti* collected in urban areas of the state of Rio Grande do Norte, Brazil. The transovarial transmission of dengue virus by *Aedes* mosquitoes is a well documented phenomenon in endemic areas worldwide (S1 Table). In this study, DENV-4 was identified in 4 pools of *A*. *aegypti* larvae/pupae with an infection rate of 3.37, a similar value found by Rohani et al. [27] in Malaysia (MIR = 3.9–100), and by Gunther et al. [12] in Mexico (MIR = 4.6), both in *A*. *aegypti*.

The presence of *A*. *albopictus* was identified in four urban areas in Natal: Felipe Camarão, Nova Descoberta, Potengi and Quintas. This may be explained by the environmental characteristics of the study site. Natal contains a large preservation area of wooded dunes that is a remnant of the Atlantic Forest ecosystem in Brazil called the "Dunes State Park" (05 ° 46'S, 35 ° 12'W). This park is crucial for water supply, outdoor activity and cultural activity. It encompasses 1,172.8 ha (15 x 2 km) and borders the Atlantic Ocean along its east bank andthe urban area of Natal along the west side of the park. Tropical fauna includes birds, marsupials, and marmosets. There are more than 350 species of native flora including trees, shrubs, bromeliads, orchids and grasses [28]. The proximity of the Dunas State Park to the urban area can promote adequate environmental conditions for the circulation of *A*. *albopictus* in both areas. No DENV was found in *A*. *albopictus* larvae. In contrast, a study conducted in Malaysia showed that 18/363 (4.9%) of larvae pools from *A*. *albopictus* were positive for dengue virus [27].

This study allowed the detection of dengue virus type 4 during the study period. A previous study conducted in the same geographic region in 2011showed DENV-1, DENV-2 and DENV-4 co-circulating, thus highlighting the important introduction of DENV-4 in the State of Rio Grande do Norte in May 2011 after a religious event held in Santa Cruz, a municipality located 100 km from Natal. DENV-4 was detected in Natal right after this event, demonstrating the speed with which these viruses can be transmitted with migratory or population movement, allowing rapid dissemination of the virus once the vectors are available. In 2012, this same study reported that the circulation of only DENV-4 was confirmed [6]. Co-circulation of serotypes 1, 2 and 4 was also shown in the years 2013 and 2014, although predominantly DENV-4 [29]. The present study reinforces the importance of applying molecular techniques such as RT-PCR in the detection of dengue virus in larvae and pupae collected in the field. Similar findings were also reported by Chao et al. [30] and Rohani et al. [27]. These techniques are practical diagnostic tools for surveillance of DENV in *Aedes* mosquitoes, serving as a warning sign during the onset of dengue transmission and could be of use to predict potential complications of multiple DENV serotypes circulating.

This work represents the most complete study to date on the interaction between dengue viruses and *A*. *aegypti* and *A*. *albopictus* mosquitoes in the State of Rio Grande do Norte, and raises important questions about a possible role of *A*. *albopictus* in the transmission of dengue virus in Brazil. Currently, the vector-fighting strategy in Brazilhas been mainly focused on *A*. *aegypti*; however, few studies have been conducted to know the role of other species in dengue transmission.

It was possible to identify three dengue serotypes in *A*.*aegypti* and *A*. *albopictus*adults: DENV-1, 2 and 4, with DENV-4 being the most prevalent, being identified in 18.4% of the studied pools. DENV-2 was only identified in *A*. *aegypti* adults. DENV-1 and DENV-4 were identified in *A*. *aegypti* and *A*. *albopictus* adults. Despite this important finding, it was not possible to evaluate the vector capacity of the investigated mosquito species. Thus, additional studies should be performed for viral identification in the salivary gland of the mosquito.

The co-circulation of three of the four dengue serotypes (DENV-1, DENV-2 and DENV-4) was clearly demonstrated in our study. This finding is in fact concerning, and should serve as an alert to health authorities for the possibility of co-infection events by more than one serotype, and the current problem is further aggravated by the co-circulation of dengue, zika and chikungunya viruses in Brazil.

In addition, the minimum infection rate in adults of the *Aedes* genus was 19.8, considering both species. Considering only the *A*.*aegypti* species, the MIR was 16.2. In analyzing the infection individually by DENV-1, DENV-2 and DENV-4 in *A*.*aegypti*, the MIR was 0.7, 0.7 and 14.6, respectively. Similar minimal infection rates were found in *A*. *aegypti* by Urdaneta et al. [31] in Venezuela (MIR = 15.9), and Garcia-Rejon et al. [32] in Mexico (MIR = 18) (S2 Table). It was not possible to calculate the MIR of the *A*. *albopictus* species, since less than 1000 individuals of this species were collected.

In conclusion, this study reveals the importance of arbovirus research in *A*. *aegypti*, *A*. *albopictus* and other species. The early detection of these viruses in vectors during entomological and virological surveillance could allow for more intense targeted control actions.

## Supporting information

S1 Fig Number of reported dengue cases (A) and fatal dengue cases (B) in Natal, Rio Grande do Norte, Brazil (2011–2014). (TIF)

S1 Table Minimum infection rate (MIR) for dengue virus in *Aedes aegypti* larvae or pupae collected in the field reported in different studies. Updated from Guedes et al. (2010). NI = Not informed in the paper. ^a^The authors do not make clear whether DENV-1 was found in *A*. *aegypti*, but mention that it was detected in *A*. *albopictus*. NS = Not specified. ^b^Memorias do Instituto Oswaldo Cruz. 2005; 100: 833–839. ^c^Proc ASEAN Congress of Tropical Medicine and Parasitology. 2008; 3: 84–89. ^d^PLoS One. 2012; 7: e41386. ^e^Revista de SaúdePública. 2008; 42: 986–991. ^f^Brazilian Journal of Biology. 2009; 69: 123–127. ^g^Dengue Bulletin. 2005; 29: 106–111. ^h^Tropical Medicine & International Health. 2004; 9: 41–46.
(DOCX)

S2 Table Minimum infection rate (MIR) for dengue virus in *A*. *aegypti* adults collected in the field reported in different studies. Updated from Guedes et al. (2010). ^a^Memórias do Instituto Oswaldo Cruz. 200297: 799–800. ^b^Tropical Medicine & International Health. 2002; 7: 322–330.
(DOCX)
