# Supplementary figures and images for: Dengue virus in Aedes aegypti and Aedes albopictus in urban areas in the state of Rio Grande do Norte, Brazil: Importance of virological and entomological surveillance
Source: PLoS One. 2018 Mar 13;13(3):e0194108. doi: 10.1371/journal.pone.0194108 (PMC5849307; doi:10.1371/journal.pone.0194108)

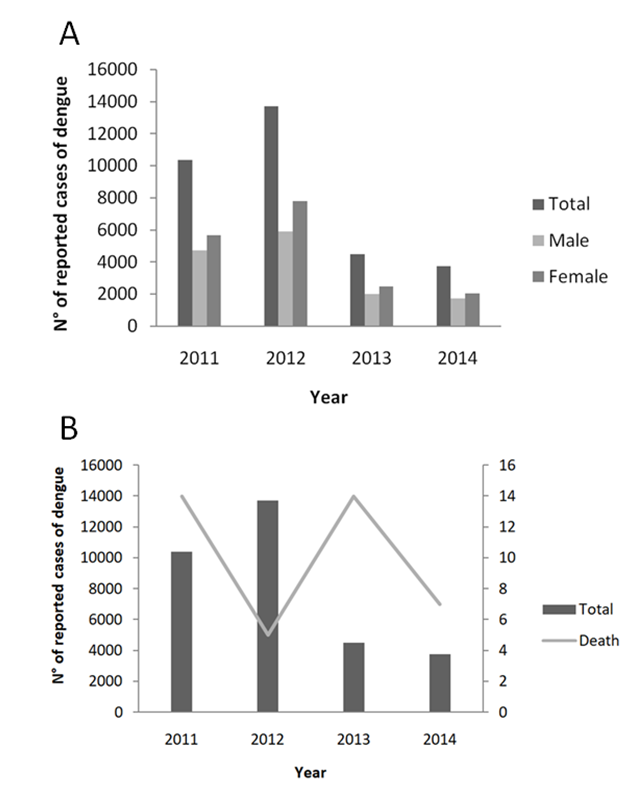

Supplement: S1 Fig — (TIF) [file pone.0194108.s001.tif]
