# Supplementary material for: Dengue virus in Aedes aegypti and Aedes albopictus in urban areas in the state of Rio Grande do Norte, Brazil: Importance of virological and entomological surveillance
Source: PLoS One. 2018 Mar 13;13(3):e0194108. doi: 10.1371/journal.pone.0194108 (PMC5849307; doi:10.1371/journal.pone.0194108)
Supplement: S1 Table — Updated from Guedes et al. (2010). NI = Not informed in the paper. aThe authors do not make clear whether DENV-1 was found in A. aegypti, but mention that it was detected in A. albopictus. NS = Not specified. bMemorias do Instituto Oswaldo Cruz. 2005; 100: 833–839. cProc ASEAN Congress of Tropical Medicine and Parasitology. 2008; 3: 84–89. dPLoS One. 2012; 7: e41386. eRevista de SaúdePública. 2008; 42: 986–991. fBrazilian Journal of Biology. 2009; 69: 123–127. gDengue Bulletin. 2005; 29: 106–111. hTropical Medicine & International Health. 2004; 9: 41–46. (DOCX) [file pone.0194108.s002.docx]

**S1 Table. Minimum infection rate (MIR) for dengue virus in *Aedes aegypti* larvae or pupae collected in the field reported in different studies.** Updated from Guedes et al. (2010). NI= Not informed in the paper. ^a^The authors do not make clear whether DENV-1 was found in *Aedes aegypti*, but mention that it was detected in *Ae. albopictus*. NS= Not specified. ^b^Memorias do Instituto Oswaldo Cruz. 2005; 100: 833–839. ^c^Proc ASEAN Congress of Tropical Medicine and Parasitology. 2008; 3: 84-89. ^d^PLoS One. 2012; 7: ^e^41386. ^e^Revista de SaúdePública. 2008; 42: 986–991. ^f^Brazilian Journal of Biology. 2009; 69:123–127. ^g^Dengue Bulletin. 2005; 29: 106-111. ^h^Tropical Medicine & International Health. 2004; 9: 41–46.

| **Stage of**  ***Ae. aegypti* collected in the field** |  | **Stage of *Ae. aegypti* assayed for DENV** |  | **MIR** | **DENV** | **Country** | **Period** | **Reference** |
| --- | --- | --- | --- | --- | --- | --- | --- | --- |
| Larvae and pupae |  | Larvae and pupae |  | 3.37 | 4 | Brazil | Apr 2011 - Mar 2012 | This study |
| Larvae |  | Larvae |  | 0.48 | 2 | Burma | Sep 1978 - Jul 1980 | [11] |
| Larvae |  | Larvae |  | 0 | - | Singapore | Apr 1995 - Jul 1996 | [26] |
| Larvae |  | Larvae |  | 0 | - | Brazil | Feb - Jun 2003 | [b] |
| Larvae |  | Larvae |  | 0 | - | Mexico | Jan - Dec 2005 | [12] |
| Larvae |  | Larvae |  | 3.9 - 100 | 1, 3 | Malaysia | NI | [27] |
| Larvae |  | Larvae and Adults |  | NS | 2 | Indonesia | Sep 2000 - Mar 2001 | [c] |
| Larvae and pupae |  | Adult females |  | 0.5 | 2, 3 | Brazil | Mar 2007 - Jul 2009 | [d] |
| Eggs |  | Larvae |  | 0 | - | Brazil | Nov 2006 - May 2007 | [e] |
| Eggs |  | Larvae |  | 18.3 | 2^a^ | Brazil | May 2003 | [f] |
| Eggs |  | Larvae |  | 5.77 - 40.0 | NS | Malaysia | Aug 1996 - Dec 1997 | [g] |
| Larvae |  | Adults males and females |  | 0.12 | 2 | Burma | Sep 1978 - Jul 1980 | [11] |
| Eggs, larvae, adults |  | Adults males and females |  | 0.36 | 4 | French Guiana | Oct 1993 - Sep 1995 | [h] |
| Larvae |  | Adult females |  | 4.6 | 2, 3, 4 | Mexico | Jan - Dec 2005 | [12] |
