# Supplementary material for: Dengue virus in Aedes aegypti and Aedes albopictus in urban areas in the state of Rio Grande do Norte, Brazil: Importance of virological and entomological surveillance
Source: PLoS One. 2018 Mar 13;13(3):e0194108. doi: 10.1371/journal.pone.0194108 (PMC5849307; doi:10.1371/journal.pone.0194108)
Supplement: S2 Table — Updated from Guedes et al. (2010). aMemórias do Instituto Oswaldo Cruz. 200297: 799–800. bTropical Medicine & International Health. 2002; 7: 322–330. (DOCX) [file pone.0194108.s003.docx]

**S2 Table. Minimum infection rate (MIR) for dengue virus in *Aedes aegypti* adults collected in the field reported in different studies.** Updated from Guedes et al. (2010). ^a^Memórias do Instituto Oswaldo Cruz. 200297:799-800. ^b^Tropical Medicine & International Health. 2002;7:322-330.

| **Stage**  **collected in the field** | **Stage assayed for DENV** | **Species** | **MIR** | **DENV** | **Country** | **Period** | **Reference** |
| --- | --- | --- | --- | --- | --- | --- | --- |
| Adult females | Adult females | *Aedes aegypti* | 16.2 | 1, 2, 4 | Brazil | May/11 - Apr/14 | **This study** |
| Adult females | Adult females | *Aedes aegypti* | 8.5 | 2 | Brazil | Jul/00 - Jun/01 | [a] |
| Adult females | Adult females | *Aedes aegypti* | 56.2 | 1, 2, 3 | Singapure | Apr/95 - Jul/96 | [26] |
| Adult females | Adult females | *Aedes aegypti* | 69 | 1, 2, 3, 4 | Singapure | Apr/97 - Dec/00 | [b] |
| Adult females | Adult females | *Aedes aegypti* | 15.9 | 1, 3, 4 | Venezuela | Nov/00 - Dec/01 | [31] |
| Adult females | Adult females | *Aedes aegypti* | 18 | 1, 2, 3 | Mexico | Mar/07 - Fev/08 | [32] |
| Adults males and females | Adult males and females | *Aedes aegypti* | 29.9 | 1, 2, 3 | Brazil | Jan/05 - Jun/06 | [13] |
| Larvae | Adult females | *Aedes aegypti* | 4.6 | 2, 3, 4 | Mexico | Jan - Dec/05 | [12] |
